# Pharmacological Treatment for Long-Term Patients with Schizophrenia and Its Effects on Sleep in Daily Clinical Practice: A Pilot Study

**DOI:** 10.3390/medicines5020044

**Published:** 2018-05-12

**Authors:** Peggy Bosch, Sabina Lim, Heike Staudte, Sujung Yeo, Sook-Hyun Lee, Pia Barisch, Benoît Perriard, Maurits Van den Noort

**Affiliations:** 1 Psychiatric Research Group, LVR-Klinik Bedburg-Hau, 47511 Bedburg-Hau, Germany; Heike.Staudte@lvr.de; 2 Donders Institute for Brain, Cognition and Behaviour, Radboud University, 6525 Nijmegen, The Netherlands; 3 Research Group of Pain and Neuroscience, College of Korean Medicine, Kyung Hee University, Seoul 130-701, Korea; sh00god@khu.ac.kr (S.-H.L.); info@mauritsvandennoort.com (M.V.d.N.); 4 College of Korean Medicine, Sang Ji University, Wonju 26339, Korea; pinkteeth@hanmail.net; 5 Institute of Experimental Psychology, Heinrich Heine University, 40225 Düsseldorf, Germany; Pia.Barisch@uni-duesseldorf.de; 6 Department of Medicine, Neurology, University of Fribourg, 1700 Fribourg, Switzerland; benoit.perriard@unifr.ch; 7 Brussels Institute for Applied Linguistics, Vrije Universiteit Brussel, 1050 Brussels, Belgium

**Keywords:** schizophrenia, pharmacological treatment, sleep, sleep disturbances, sleep disorders, gender

## Abstract

**Background:** Pharmacological treatment is still the key intervention in the disease management of long-term patients with schizophrenia; however, how it affects sleep and whether gender differences exist remains unclear. **Methods:** Forty-six long-term outpatients with schizophrenia entered the study. The numbers of antipsychotics, sleep medications, antidepressants, and anxiolytics were analyzed. Moreover, all patients were tested using the Pittsburgh Sleep Quality Index (PSQI) and the Epworth Sleepiness Scale (ESS). Correlation analyses were conducted between the medication used and the scores on the two subjective sleep inventories. **Results:** A large variability, ranging from 0 to 8, in the total number of psychiatric drugs per person was found between the patients. Despite ongoing pharmacological treatment, the patients scored high on the PSQI, but not on the ESS; this indicates that they report problems with sleep, but not with daytime sleepiness. A significant positive correlation between the use of antipsychotics and the ESS score, but not the PSQI score, was found; moreover, no gender differences were found. **Conclusions:** A large variability exists in the pharmacological treatment of long-term patients with schizophrenia. To date, patients’ sleep problems have been insufficiently treated, and gender differences have not been adequately accounted for in the pharmacological treatment of schizophrenia. More and larger international clinical studies are warranted to verify the findings of the present preliminary pilot study before any firm conclusions can be drawn and before any changes to the drug treatment of male and female patients with schizophrenia can be recommended.

## 1. Introduction

Pharmacological treatment is still the key intervention in the disease management of patients suffering from schizophrenia [1]. Antipsychotics are mostly prescribed to patients by their psychiatrists [2]. A distinction is made between the so-called first-generation antipsychotics that were developed in the 1950s and the second-generation antipsychotics that were developed in the 1980s [3]. Examples of first-generation antipsychotics (sometimes referred to as conventional antipsychotics) are haloperidol [4], chlorpromazine [5], fluphenazine [6], thiothixene [7], and perphenazine [8], whereas examples of second-generation antipsychotics (sometimes called atypical antipsychotics) commonly prescribed in the treatment of schizophrenia are risperidone [9], olanzapine [10], quetiapine [11], aripiprazole [12], and asenapine [13].

Despite the pharmacologic advances in the treatment of patients with schizophrenia, important issues still exist and need to be addressed in order to optimalize individual treatment [14]. One of these issues is the large interindividual differences in the ways in which patients with schizophrenia respond to antipsychotics [15]. Moreover, the optimal dose of a specific antipsychotic differs markedly between patients, probably due to the patient’s unique genetic markers [15]; this needs to be considered when developing an optimal treatment for each individual [16]. Both first- and second-generation antipsychotics have important side effects that again differ between patients to a large extent [15,17]. Previous research showed that first-generation antipsychotics have higher rates and risks of extrapyramidal side effects than second-generation antipsychotics [18]. Second-generation antipsychotics were developed in order to overcome those extrapyramidal side effects, and although they partially succeeded [18], second-generation antipsychotics were unfortunately found to have higher risks of causing metabolic side effects [19], weight gain [19], and cardiovascular problems [20]. Furthermore, they were unable to completely overcome the extrapyramidal side effects [2,18]. To sum up, first-generation and second-generation antipsychotics are different in the ways they treat the positive, negative, and cognitive symptoms of patients suffering from schizophrenia. With respect to the efficacy on the individual level, large differences exist between persons; sometimes first-generation antipsychotics are more efficient than second-generation antipsychotics and vice versa [21]. Moreover, none of the abovementioned drugs has been found to be the gold standard, and although monotherapy is recommended [22], polypharmacy is often used in daily clinical practice [22].

Besides the side effects mentioned above, antipsychotics also have an effect on the co-morbid sleep disorders that often exist in patients with schizophremia [23]. Patients with schizophrenia mostly show a longer sleep latency, a decrease in total sleep time, and a decrease in sleep efficiency [24]. The underlying sleep architecture of patients with schizophrenia revealed reductions in non-rapid-eye-movement sleep, slow-wave sleep, and rapid-eye-movement sleep latency [24]. In addition, rapid-eye-movement sleep duration was found to remain stable in patients with schizophrenia [25]. Although the side effects of the medication can sometimes be beneficial [26], i.e., the patients start to sleep better [26], they can also be negative [26], i.e., the patients sleep worse [26]. So far, with respect to the effects of antipsychotics on sleep, it is known from electroencephalographic sleep studies on healthy participants that the use of antipsychotic drugs, such as clozapine [27], olanzapine [28], quetiapine [29], and ziprasidone [30], leads to an augmentation of sleep efficiency and/or total sleep time [24,27,28,29,30]. In addition, previous research showed that olanzapine [28] and ziprasidone [30] increased slow-wave sleep, but mixed results were found with respect to the rapid-eye-movement results [24]. In patients with schizophrenia, it was found that the use of clozapine, olanzapine, and paliperidone in most cases resulted in a significantly shorter sleep latency and a significantly longer total sleep time as well as increased sleep efficiency [24]. Moreover, olanzapine [31] and paliperidone [32] increased both slow-wave sleep and rapid-eye-movement sleep. In addition, quetiapine was found to have a negative influence on sleep and resulted in a longer sleep latency, wake time after sleep onset, and rapid-eye-movement sleep latency, as well as in a decrease of slow-wave sleep and rapid-eye-movement sleep [33]. With respect to the effects of risperidone on sleep, the results are as yet inconclusive [24]. Finally, it is important to note that so far, for several antipsychotics, their effects on sleep in healthy individuals or patients with schizophrenia are either insufficiently investigated and/or completely unknown.

Although sleep disorders are increasingly recognized as a major problem for patients suffering from schizophrenia [34] and normalized sleep has been found to be vital for a good clinical outcome [35], in most cases, quality of sleep is not the direct focus of treatment [34]. Patients with schizophrenia and co-morbid sleep disorders often receive antipsychotics, as well as pharmacological treatment for their sleep disorders. Here, one can think of the following sleep medications that are often prescribed: zopiclone [36], phenobarbitone [37], zolpidem [37], diazepam [37], etc. Unfortunately, those sleep medications interact with the pharmacological treatment of the positive, negative, and cognitive symptoms, which is the reason why determining optimal individual pharmacological treatments for such patients is difficult. Pharmacological guidelines for treatment have been developed [38], but recommendations vary in specificity [38]. Interestingly, when writing guidelines for the treatment of these patients, concerns about side effects were found to be more important than the efficacy of the medication [38]. Moreover, the choice and the dosage of the antipsychotic, the duration of its use, and the way to handle treatment resistance should be investigated, as they have been found to be often underestimated [38].

During the last decade, gender differences have finally received more attention in pharmacological treatment [39]. Research has shown sex to be a risk factor regarding the side effects of medications [39]. However, most studies on antipsychotics still do not address the effects of the many potential gender differences (e.g., differences in hormones, changes in hormone production throughout a women’s life, etc.) on the efficacies of medications, the doses of medications, and other gender-specific considerations [40]. At present, many gender issues are still under-investigated or not investigated at all, and if a more optimal pharmacological treatment of patients with schizophrenia and co-morbid sleep disorders is to be found, this gap of missing knowledge needs to be closed [40]. The question arises as to whether gender differences can be found in practice when it comes to the antipsychotics that are used.

The aim of the present study is to investigate the pharmacological treatment of long-term outpatients with schizophrenia, how that treatment affects the subjective quality of sleep, and whether gender differences exist. First, a large variability is expected to be found in the pharmacological treatment of long-term patients with schizophrenia because, from the literature, patients with schizophrenia are known to comprise a very heterogeneous clinical patient group. Second, based on previous research, long-term patients with schizophrenia are hypothesized to suffer from sleep problems. Third, an interaction between the pharmacological treatment and the subjective quality of sleep is expected to exist; the more antipsychotic medications a long-term patient with schizophrenia receives, the more daytime drowsiness will be reported. Our fourth hypothesis is that gender differences will not be found in the pharmacological treatment regarding the numbers of drugs the patients have been prescribed because, from the literature, gender differences are known to be often unaccounted for in clinical research and daily clinical practice. Finally, the implications of our results will be discussed and recommendations will be made for future research, as well as for clinical practice.

## 2. Materials and Methods

### 2.1. Participants

Forty-six long-term outpatients with schizophrenia were included in the study. They all signed informed consent and their psychiatrists confirmed that they were able to do so. All patients with schizophrenia were diagnosed by their psychiatrists in the clinic in accordance with The ICD-10 Classification of Mental and Behavioural Disorders: Clinical Descriptions and Diagnostic Guidelines [41]. The patients included 20 males and 26 females. The patients had a mean length of illness of 10.08 (*SD* = 5.25) years and a mean age of onset of disease of 32.75 (*SD* = 9.20) years. The male patients had a mean age of 42.20 (*SD* = 9.49) years, and the female patients had a mean age of 43.31 (*SD* = 10.39) years. Because previous research found important age-related differences in quality of sleep [42], testing for such differences was important. The Analysis of Variance (ANOVA), however, showed no significant difference in age between the male and the female groups (*F*_(1,45)_ = 0.138, *p* = 0.712, *η*^2^ = 0.003) in this study. Moreover, an ANOVA was conducted in order to test whether differences in intelligence existed between the two groups, as intelligence might be a factor that needs to be controlled in order to make the self-rating results of male and female patients with schizophrenia on self-report questionnaires more reliable and comparable [43]. Intelligence was measured using the Mehrfach-Wortschatz-Intelligenz-Test-B (MWT-B) [44]. The ANOVA results proved that no significant difference in the mean intelligence scores between the male (*M* = 101.00, *SD* = 15.65) and the female (*M* = 201.14, *SD* = 11.52) patient groups existed (*F*_(1,38)_ = 0.068, *p* = 0.795, *η*^2^ = 0.002). Exclusion criteria were other (besides the medications that are part of the individual pharmacological treatment, caffeine, and/or nicotine use) addictive soft and hard drugs use, such as cannabis, marijuana, cocaine, heroin, etc., and/or extensive alcohol use. All patients participated on a voluntary basis, and none received a financial reward. The clinical study was approved by the local ethics committee called Ärztekammer Nordrhein (under number: 2008331), and the clinical trial was officially registered at the Dutch Trial Register (under number NTR3132/see also http://www.trialregister.nl/trialreg/admin/rctview.asp?TC=3132). Finally, the Declaration of Helsinki (http://www.wma.net/en/30publications/10policies/b3/) was followed during the conduct of the study.

### 2.2. Pharmacological Data

The total numbers of medications, as well as the total numbers of antipsychotics, specific sleep medication, evening sleep-enhancing medications, antidepressants, anxiolytics, and other medications, were analyzed per patient. These data were available from a clinical database. The pharmacological data for the participating outpatients was taken at the moment they entered the study.

### 2.3. Sleep Inventories

Two subjective sleep questionnaires were included in the study: The Pittsburgh Sleep Quality Index (PSQI) [45] and the Epworth Sleepiness Scale (ESS) [46]. The PSQI [45] is a self-report instrument with questions related to the individual’s sleep over the last four weeks. The instrument consists of 18 items and gathers information on the number of sleep disturbances, estimates on sleep quality, sleep duration, sleep latency and sleep times, use of medication, and sleepiness during the day. The items are summarized into seven components, each of which receives a score between 0 and 3. As a result, the total score is a summation of the scores on those seven components and lies between 0 and 21. The higher the score, the poorer the individual’s sleep quality [45].

The ESS [46] is a self-report instrument consisting of eight questions. Individuals need to rate, on a four-point scale (ranging from 0 = will never fall asleep to 3 = high probability of falling asleep), the chances of dozing off or falling asleep while conducting eight different activities. Most individuals participate in those eight activities on a regular basis; they do not need to be engaged in those activities on a daily basis (examples are watching television, sitting in a room talking to somebody, etc.). The ESS score is a summation of the scores on the eight items, and as a result, the ESS score lies somewhere between 0 and 24. A high ESS score means a high ”daytime sleepiness”, and a low ESS score a low “daytime sleepiness”. A score between 11 and 15 is considered to be a “mild-to-moderate” sleep apnea while a score of 16 or above is considered to be a “severe” sleep apnea [46].

### 2.4. Procedure

The patients with schizophrenia voluntarily entered the study. First, they received general information about the study and signed informed consent forms. This was followed by more specific verbal and written instructions about the two specific sleep instruments, after which the patients completed the PSQI [45] and the ESS [46], which took approximately 15 to 25 min, in a quiet room in the clinic. A member of the staff was present in the room in case the patient had any questions. After the completion of the two sleep instruments, all patients were debriefed.

### 2.5. Statistics

Statistical Package for the Social Sciences (SPSS) version 22.0 (IBM Corp., Armonk, NY, USA, 2013) was used for all statistical analyses. An Analysis of Variance (ANOVA) was conducted to test whether differences in scores on the two subjective sleep inventories existed between the male patient group and the female patient group. Moreover, Pearson correlation analyses (two-tailed) were used to test for a possible linear relationship between the patient’s subjective quality of sleep and the patient’s pharmacological treatment. For all statistical analyses, a significance level of *p* < 0.05 was used [47].

## 3. Results

### 3.1. Total Medication Use

As can be seen in Table 1, in general, the long-term patients with schizophrenia received three medications together. No difference could be found between male and female patients regarding the mean number of medications received (*F*_(1,45)_ = 0.008, *p* = 0.929, *η*^2^ = 0.000). The total number of prescribed psychiatric medications varied between patients over a large range from zero up to even eight different prescribed medications per patient.

### 3.2. Antipsychotic Medication

In general, patients with schizophrenia received 2.07 antipsychotics in the pharmacological treatment of their positive, negative, and cognitive symptoms. Some patients received no antipsychotic medication at all, whereas other patients received up to five different kinds of antipsychotics. In general, the male patients received 2.11 antipsychotics versus 2.04 for the female patients. As can be seen in Table 1, no significant difference in the number of prescribed antipsychotics between the male and the female patients with schizophrenia was found (*F*_(1,45)_ = 0.025, *p* = 0.875, *η*^2^ = 0.001). The most frequently prescribed antipsychotics were quetiapine (13/46 = 28.26%), risperidone (13/46 = 28.26%), and olanzapine (8/46 = 17.39%).

### 3.3. Sleep Medication

A close look at the use of sleep medication shows that only two patients (2/46 = 4.35%) received a specific sleep medication; in both cases, the patients were male. However, an analysis of the male and the female groups together showed no significant difference in sleep medication use (*F*_(1,45)_ = 2.692, *p* = 0.108, *η*^2^ = 0.062) (see Table 1). The sleep medication that was prescribed was zopiclone (2/46 = 4.35%). In most cases (39/46 = 84.78%), however, patients received other medications (often antipsychotics) as a sleep-ameliorating drug in the evening. The frequency of this clinical pharmacological intervention to improve sleep quality by using a non-specific sleep medication was the same in the male and the female patients with schizophrenia (*F*_(1,45)_ = 0.006, *p* = 0.940, *η*^2^ = 0.000) (see also Table 1).

### 3.4. Additional Medications

As can be seen in Table 1, in addition to the prescription of antipsychotics and sleep medications, some patients with schizophrenia were prescribed antidepressants and anxiolytics. Again, the male patients did not differ from the female patients regarding the use of antidepressants (*F*_(1,45)_ = 0.522, *p* = 0.474, *η*^2^ = 0.013) or anxiolytics (*F*_(1,45)_ = 2.588, *p* = 0.115, *η*^2^ = 0.059).

### 3.5. Subjective Quality of Sleep Measurements

With respect to the scores on the two subjective sleep quality inventories, firstly, as can be seen in Table 2, no significant difference in the mean total PSQI scores between the male patient group and the female patient group was found (*F*_(1,43)_ = 0.865, *p* = 0.358, *η*^2^ = 0.020). Both the male patient group and the female patient group scored above the generally used cut-off score of 5 [45], as well as the more recently used stricter cut-off score of 6 [48]. Two patients with schizophrenia (2/46 = 4.35%) were unable to complete the PSQI. Secondly, no significant difference in the mean total ESS scores between the male patient group and the female patient group was found (*F*_(1,44)_ = 0.119, *p* = 0.732, *η*^2^ = 0.003). Both the male patient group and the female patient group scored below the generally used cut-off score of 9 [46]. One patient with schizophrenia (1/46 = 2.17%) was unable to complete the EES.

### 3.6. Interaction Effects between Pharmacological Treatment and Quality of Sleep

Correlation analyses on the medications used and the PSQI scores, as well as on the medications used and the ESS scores, were conducted. The Pearson correlation analysis on the medications used and the PSQI scores revealed no significant correlations between the number of antipsychotics and the scores on the PSQI (*r* = −0.145, *p* = 0.364), the number of specific sleep medications and the scores on the PSQI (*r* = 0.163, *p* = 0.310), or the total number of medications and the scores on the PSQI (*r* = 0.025, *p* = 0.878). As to the Pearson correlation results for the medications used and the EES scores, a significant positive correlation was found to exist between the number of antipsychotics and the score on the ESS (*r* = 0.354, *p* = 0.021), but no significant correlations were found between the number of specific sleep medications and the ESS scores (*r* = −0.201, *p* = 0.203) or the total number of medications and the ESS scores (*r* = 0.252, *p* = 0.108).

## 4. Discussion

The aim of the present study was to investigate the pharmacological treatment of long-term outpatients with schizophrenia, how that treatment affects the subjective quality of sleep, and whether gender differences exist. First, we analyzed the pharmacological treatment of the patients. The results clearly proved our expectation that a large variability existed in the pharmacological treatment of long-term patients with schizophrenia. While some patients received no antipsychotics at all, others received up to five different kinds of antipsychotics. Furthermore, the total number of psychiatric medications ranged from zero up to even eight different medications. This finding stresses the importance of both the implications for a patient of taking eight different medications each day and the problem of all the different side effects, which are largely unknown, interacting with one another [49]. Moreover, cost-effectiveness studies on the efficiency of antipsychotic polytherapy versus antipsychotic monotherapy for long-term patients with schizophrenia are lacking so far [50]. Those are needed in the future in order to gain scientific proof for the often preferred antipsychotic polytherapy in daily clinical practice, which was also found to be preferred in the present study and which is, in fact, contrary to the official recommendation of antipsychotic monotherapy for the treatment of patients with schizophrenia [22,51]. Our pharmacological data clearly illustrate the problem of heterogeneity within the group of long-term patients with schizophrenia. Moreover, these data further stimulate the ongoing clinical discussion of whether diagnostically one patient group with schizophrenia or all kinds of groups with different subtypes of schizophrenia exist [52], as was described in the previous versions of the Diagnostic and Statistical Manual of Mental disorders (DSM) classification system (e.g., DSM-IV [53], DSM-IV-TR [54]).

Secondly, we were interested in the question of whether our clinical sample of long-term outpatients with schizophrenia suffered from sleep problems. We expected that this would, indeed, be the case. If we look at our data, the results of the subjective sleep quality inventories show that the patients scored above the generally used cut-off score of 5 [45], as well as above the more recently used stricter cut-off score of 6 [48], meaning that they are poor sleepers. Here, noting that these patients scored above the cut-off scores despite their ongoing pharmacological treatment is important. Probably, without this pharmacological treatment, particularly their specific sleep medication and the medication that enhanced their sleep, their sleep problems would have been even worse. Moreover, this finding shows that the ongoing pharmacological treatment is unable to adequately treat the sleep problems in this patient group and that further improvements in the disease management of the co-morbid sleep disorders are needed. With respect to the second subjective sleep inventory that measured daytime sleepiness, the long-term patients with schizophrenia scored below the generally used cut-off score of 9 [46], meaning that they did not suffer from daytime sleepiness in comparison with the healthy norm. With respect to daytime sleepiness, the ongoing pharmacological treatment of the patients did not seem to increase their sleepiness, as might have been expected, because sleepiness has often been mentioned as a side effect [55,56]. Nevertheless, in the future, the pharmacological treatment may be further improved so that long-term male and female patients with schizophrenia, on average, score close to 0 on the ESS instead of around the value of 6 at present. Moreover, noting that almost one out of three (14/45 = 31.11%) of our patients still reported suffering from daytime sleepiness (ESS scores > 9), which is a significant number, is important.

Our third hypothesis was that an interaction between the pharmacological treatment and the subjective quality of sleep was expected to exist. Overall, the results from this study regarding the correlation between the pharmacological treatment and the subjective quality of sleep are mixed, depending on whether the focus is on the broad spectrum of sleep problems, as measured with the PSQI [45], or on daytime sleepiness specifically, as measured with the ESS [46]. First, when looking at the PSQI results [45], no correlation between the pharmacological treatment and the scores on the PSQI was found. This can be explained as follows: Some patients seem to benefit from the use of drugs [26], whereas other patients have negative sleep effects due to the pharmacological treatment [26]. When all pharmacological data are taken together, the positive and the negative effects of the use of drugs seem to counterbalance, so no significant positive or negative correlation is found. Our findings are in line with the mixed findings that have been previously reported in the literature [26]. For instance, the first-generation antipsychotics haloperidol, thiothixene, and flupentixol were found to significantly reduce stage-two sleep latency and significantly increase sleep efficiency, whereas the second-generation antipsychotics olanzapine, risperidone, and clozapine were found to significantly increase both total sleep time and stage-two sleep [57]. However, our expections were fulfilled by our data when looking at the daytime sleepiness results specifically. A positive relation was found between the number of antipsychotic medications the patient used and the daytime drowsiness the patient reported on the ESS [46]. The more antipsychotic medications the patient used, the more daytime drowsiness the patient experienced.

An important difference exists between the ESS [46] and the PSQI [45]. The PSQI focuses on the overall quality and quantity of sleep, as well as general sleepiness. The results show that those components seem to be altered in our subjects, no matter how many antipsychotics they take. Those patients suffer from a generally dimished quality and quantity of sleep compared to the healthy population, irrespective of the pharmacological treatment they receive. In contrast, the ESS measures sleepiness while carrying out different activities during the day. Here, a higher number of antipsychotics was found to go together with more drowsiness while doing those activities. Thus, while the long-term patients with schizophrenia do generally show poorer quality of sleep, the number of antipsychotics taken especially has a negative effect on daytime alertness and associated attention and concentration. Due to the lack of alertness, those patients show an increased drowsiness and faster exhaustion during daily activities. However, these are only speculations. If the sleep problems in long-term patients withschizophrenia and the way in which pharmacological treatment affects the quality of sleep in such patients are to be better understood, further investigations of exactly which aspects of sleep are affected by the drugs are needed. Based on such knowledge, individual treatment may be further improved.

Our fourth hypothesis was that gender differences will not be found in the pharmacological treatment of long-term patients with schizophrenia because, according to the literature, such differences are known to be often unaccounted for in clinical research [58] and in daily clinical practice [58]. The results of our study are clearly in line with our expectations. No significant differences between the pharmacological treatments of males and females were found for the numbers of antipsychotics, sleep medications, antidepressants, and anxiolytics. As already mentioned in the introduction, good reasons exist for considering the gender of the patient when choosing the pharmacological treatment [59]. Research has shown that gender is a risk factor for side effects from certain medications [60]. Female patients were found to have at least a 1.5-fold greater risk of developing an adverse drug reaction than male patients [61]. Thus, one drug or a combination of different medications can have fundamentally different effects on male and female patients. However, as our studies found, gender seems to be forgotten when prescribing medicines; thus, one can conclude that the side effects suffered by patients can be reduced by taking into account the gender when planning the optimal pharmacological treatment for the individual patient. In the future, this gap of knowledge needs to be closed; studying the differences in the efficiencies of the medications between male and female patients with schizophrenia is vital if the side effects of different drugs and the interactions between different drugs are to be better controlled.

An important difference seems to exist between research and clinical practice with respect to the pharmacological treatment of patients with schizophrenia and co-morbid sleep disorders, and this difference needs to be eliminated. Although monotherapy is recommended [22], in daily clinical practice, this is more the exception than the rule [22]. Patients often receive all kinds of antipsychotics and sleep medications in order to deal with their positive, negative, and cognitive symptoms. Therefore, many results obtained in scientific research are not directly transferrable to clinical practice.

A limitation of the present study is that the data were gathered in only one German psychiatric clinic. As such, the general implications of our clinical study are limited, and it should be considered as a pilot study. More data from other German psychiatric clinics, as well as larger international multi-center studies, are needed in order to be able to draw firm conclusions that have more general implications. For instance, different dosing of drugs for men and for women might be necessary. As an example, in the specific case of zolpidem, The United States Food and Drug Administration required labeling changes on 19 April 2013 [62]. It had previously been found that women have higher morning serum zolpidem concentrations than men after taking an evening dose and this could lead to higher risks of harm [63]. The present clinical study, however, was not large enough to suggest such changes. Nevertheless, despite this limitation, the present study addresses several important issues, such as the general importance of gender differences, which should be better taken into account in the study of the clinical pharmacological treatment of patients with schizophrenia and co-morbid sleep disorders.

Another limitation of the current study is that some of the long-term patients with schizophrenia were using caffeine and/or nicotine besides their pharmacological treatment. Previous research has shown that caffeine [64] and nicotine [65] have a disruptive effect on quality of sleep. In order to keep our sample as close as possible to the daily clinical routine, it was chosen not to ask the patients about their caffeine and/or nicotine consumption. The idea behind this was that such questioning could have influenced the behavior of our patients because by asking them and making them alert of their caffeine and/or nicotine consumption one uses a kind of clinical intervention. On the other hand, the amount of caffeine and/or nicotine use of the individual patients could have been used as an important and interesting covariate in our analyses of the relation between pharmacological treatment and the quality of sleep as well as possible gender differences. For instance, previous research found that differences in serum levels between male and female smokers exist after controlling for the amount of nicotine [66].

A third limitation of our study is that we focused only on long-term patients with schizophrenia. In future research, including first-episode patients with schizophrenia as well would be interesting [67]. This would give better insight into the sleep problems experienced during the acute phase of psychosis. For instance, collecting subjective [68] and objective [68] sleep data before any pharmacological treatment has started and comparing these sleep data with the results after the pharmacological treatment has started and at follow-up would be highly informative. Sleep problems seem to be an important early warning sign for schizophrenia [69] and might be used as a marker for psychiatrists, psychologists, nurses, etc. in clinical practice, as well as for relatives and friends in the social field of the patient, to prevent upcoming psychotic relapses.

In addition, in future research it would be interesting to collect serum levels of the medication taken [70]. In previous research, for instance, it has been observed that the serum levels of cytokine IL-3 are significantly elevated in patients with long-term schizophrenia [71]. In this study, no serum levels were collected, due to lack of funding. Moreover, it is not part of the standard care in our clinic to collect serum levels of all medication at standard intervals; serum levels are measured when the psychiatrist who treats a patient sees a necessity. For an overview of the exact medication that was prescribed, we refer to Supplementary Table 1. In future studies, it would be beneficial to collect serum levels in order to investigate the relationship between the subjective sleep assessments and the medication [72].

Finally, it would have been interesting to analyze the pharmacological data at several time points during a longer period of treatment [73]. The current study was not planned and approved for this purpose; therefore, we were unfortunately unable to do this in the present study. However, in future research, it would be highly interesting to include several measurement points [73] over a longer period (e.g., for instance, additional measurements after six months, one year, two years, and four years) since the pharmacological treatment of patients with long-term schizophrenia is not a static but a dynamic process. This could provide important data on the changes in dosage and number of medications over a longer treatment period; moreover, it could give important information about the treatment compliance of the patients [73].

## 5. Conclusions

A large variability in the pharmacological treatment of long-term patients with schizophrenia exists. To date, patients’ sleep problems have been insufficiently treated, and gender differences have not been adequately accounted for. More and larger international clinical studies are warranted to verify the findings of the present preliminary pilot study before any firm conclusions can be drawn and before any changes to the drug treatment of male and female patients with schizophrenia can be recommended.

## Figures and Tables

**Table 1:** Overview of the average numbers of medications used by male and female long-term outpatients suffering from schizophrenia.

Medication	Total	Male Group	Female Group
Total number of medications	2.98 (*SD* = 1.90)	2.95 (*SD* = 2.01)	3.00 (*SD* = 1.84)
Number of antipsychotics	2.07 (*SD* = 1.30)	2.11 (*SD* = 1.37)	2.04 (*SD* = 1.27)
Number of specific sleep medications	0.05 (*SD* = 0.21)	0.11 (*SD* = 0.32)	0.00 (*SD* = 0.00)
Number of evening sleep-enhancing medications	0.84 (*SD* = 0.37)	0.84 (*SD* = 0.37)	0.83 (*SD* = 0.38)
Number of other medications	0.33 (*SD* = 0.57)	0.37 (*SD* = 0.60)	0.29 (*SD* = 0.55)
Number of antidepressants	0.44 (*SD* = 0.59)	0.37 (*SD* = 0.60)	0.50 (*SD* = 0.59)
Number of anxiolytics	0.07 (*SD* = 0.26)	0.00 (*SD* = 0.00)	0.13 (SD = 0.34)

**Table 2:** Overview of the subjective sleep quality scores of the long-term outpatients with schizophrenia on the The Pittsburgh Sleep Quality Index (PSQI) and the Epworth Sleepiness Scale (ESS) for the total group, the male group, and the female group.

Sleep Inventories	Total	Male Group	Female Group
PSQI	8.52 (*SD* = 4.70)	7.80 (*SD* = 4.69)	9.13 (*SD* = 4.72)
ESS	6.07 (*SD* = 4.01)	6.30 (*SD* = 4.18)	5.88 (*SD* = 3.95)

## Supplementary Materials

The following are available online at http://www.mdpi.com/2305-6320/5/2/44/s1, Table S1: Overview of the medication use specified for gender, type of medications and dosage/day per long-term outpatient suffering from schizophrenia.
