# Supplementary material for: Pharmacological Treatment for Long-Term Patients with Schizophrenia and Its Effects on Sleep in Daily Clinical Practice: A Pilot Study
Source: Medicines (Basel). 2018 May 12;5(2):44. doi: 10.3390/medicines5020044 (PMC6023313; doi:10.3390/medicines5020044)
Supplement: Supplementary file 1 [file medicines-05-00044-s001.pdf]

Supplementary Materials: Pharmacological Treatment for Long-Term Patients with Schizophrenia and Its Effects on Sleep in Daily Clinical Practice: A Pilot Study

Peggy Bosch, Sabina Lim, Heike Staudte, Sujung Yeo, Sook-Hyun Lee, Pia Barisch, Benoît Perriard and Maurits Van den Noort

Table S1. Overview of the medication use specified for gender, type of medications and dosage/day per long-term outpatient suffering from schizophrenia.

Patients	Gender	Type of Medication	Daily dosage
1	Male	Lithium	400 mg
		Amitriptylin	70 mg
		Quetiapine	450 mg
		Zotepine	100 mg
		Sulpirid	50 mg
		Ziprasidone	40 mg
		Trimipramine	125 mg
2	Male	Perazine	25 mg
		Zotepine	100 mg
		Risperidone	25 mg every 14 days
		Biperiden	1 mg
3	Male	Flupentixol	10 mg
		Risperidon	4 mg
		Flupentixol depot 10% injection	1 ml every 14 days
		Promethazine	75 mg
4	Female	Zopiclone	3.75 mg
		Mirtazapine	45 mg
		Prothipendyl	40 mg
		Pipamperone	80 mg
		Risperidone	6 mg
5	Female	Fluphenazine	25 mg, every 21 days
		Olanzapine	15 mg
6	Male	Clozapine	500 mg
7	Female	Lamotrigine	100 mg
		Clozapine	125 mg
		Risperidone	2 mg
8	Female	Quetiapine	25 mg
		Clozapine	25 mg
		Haloperidol	1.5 ml every 28 days
9	Female	Chlorprothixene	50 mg
		Olanzapine	15 mg
10	Female	Pramipexole	0.70 mg
		Amisulpride	200 mg
		Olanzapine	5 mg
11	Female	Duloxetine	60 mg
		Prothipendyl	20 mg
		Ziprasidone	120 mg
12	Female	Ziprasidone	60 mg
		Quetiapine	600 mg
13	Female	Reboxetine	6 mg
		Chlorprothixene	When needed 15 mg
		Quetiapine	300 mg
		Risperidone	2 mg
		Zotepine	200 mg
		Melperone	When needed 25 mg
14	Male	Reboxetine	4 mg
		Olanzapine	15 mg
		Zotepine	75 mg

		Flupentixol	10% 1 mg every 14 days
		Lithiumcarbonat	24 mmoL
		Amisulpride	200 mg
15	Male	Risperidone	25 mg every 21 days
		Aripiprazole	10 mg
		Mirtazapine	15 mg
16	Male	Currently no medication	-
		Melperone	75 mg
17	Female	Risperidone	3 mg
		Doxepine	25 mg
		Pipamperone	80 mg
18	Male	Lithiumcarbonat	36 mmoL
		Quetiapine	500 mg
		Quetiapine	25 mg
19	Male	Doxepine	150 mg
		Zopiclone	7.5 mg
		Ziprasidone	40 mg
20	Male	Fluphenazine	25, every 14 days
		Chlorprothixene	15 mg
		Quetiapine	150 mg
		Aripiprazole	15 mg
21	Female	Trimipramine	50 mg
		Olanzapine	15 mg
22	Female	Clozapine	175 mg
		Lithium	24 mmoL
23	Female	Paroxetin	30 mg
		Clozapine	200 mg
		Amisulprid	400 mg
24	Male	Olanzapine	20 mg
		Amisulpride	800 mg
25	Male	Currently no medication	-
		Mirtazapine	30 mg
		Pregabalin	300 mg
26	Female	Risperidone	25 mg every 14 days
		Risperidone	4 mg
		Trimipramin	125 mg
		Promethazine	50 mg and 50 if needed
		Quetiapine	300 mg
		Amisulpride	200 mg
27	Male	Risperidone	37.5 mg every 14 days
		Doxepine	50 mg
		Perazine	25 mg
28	Female	Risperidone	1 mg
29	Female	Olanzapine	5 mg
		Nortriptyline	75 mg
		Doxepine	25 mg
		Pipamperone	100 mg
30	Male	Flupentixol	10% 1 ml every 14 days
		Gabapentin	600 mg
		Pipamperone	240 mg
		Amisulpride	800 mg
		Sertraline	150 mg
		Quetiapine	200 mg
		Lithium	24 mmoL
		Chlorprothixene	If needed 30 mg
31	Female	Risperidone	4 mg
		Ziprasidone	40 mg
		Mirtazapine	30 mg
		Escitalopram	20 mg
		Pipamperone	40 mg
		Quetiapine	200 mg
		Valproate	450 mg
32	Male	Pipamperone	If needed 40 mg
		Clozapine	25 mg

		Amineurin	50 mg
		Citalopram	30 mg
		Fluspirilene	1.5 mg per week
		Ziprasidone	40 mg
		Paroxetine	10 mg
		Thioridazine	500 mg
		Ziprasidone	80 mg
		Promethazine	If needed 25 mg to 50 mg
33	Female	Valproate	500 mg
		Prothipendyl	80 mg when sleepdisorders occur
		Zopiclone	If needed 7.5 mg instead of Prothipendyl
		Pipamperone	40 mg (if needed max 120 mg)
34	Female	Zotepine	50 mg
		Pregabalin	750 mg
		Trimipramine	150 mg
35	Female	Ziprasidone	120 mg
		Clozapine	475 mg
		Doxepine	100 mg
36	Male	Quetiapine	600 mg
		Risperidone	2 mg
37	Female	Citalopram	10 mg
		Aripiprazole	15 mg
		Paliperidone	75 mg once a month
		Risperidone	1 mg
		Risperidone	50 mg every 14 days
38	Female	Benperidol	6 mg
		Sertraline	50 mg
		Flupentixol	5 mg
39	Male	Paliperidone	75 mg every 28 days
40	Female	Quetiapine	300 mg
41	Male	Venlafaxine	37.5 mg
		Aripiprazole	5 mg
42	Female	Aripiprazole	10 mg
		Sertraline	150 mg
		Quetiapine	300 mg
43	Male	Risperidone	25 mg every 21 days
		Promethazine	50 mg
44	Female	Paliperidone	75 mg every 28 days
45	Female	Currently no medication	-
46	Male	Paliperidone	100 mg every 28 days
		Pipamperone	If needed 40 mg
